# 

**Published:** 1897-03

**Authors:** 


### CONTENTS.

ORIGINAL COMMUNICATIONS—

A Simple Method of Transfusion. By Hunter P. Cooper, M.D., Atlanta, Ga...... 1

Two Cases of Gunshot Wounds: One of the Axilla, in which the Subclavian Artery was Tied. The Other, of the Popliteal Space, in which the Femoral Artery was Tied in Scarpa's Triangle, with Secondary Amputation of Thigh Ten Days Later. By W. H. Hudson, M.D., LaFayette, Ala................ 4

Some Practical Thoughts on the Development of the Human Race and Obstetric Nursing. By R. R. Kime, M.D., Atlanta, Ga....... ....................... 8

Some Interesting Cases of Skin and Venereal Disease. By Bernard Wolff, M.D., Atlanta, Ga........................................................................ 19

Diastase in Therapeutics. By C. C. Fite, M.D., New York........................ ... 24

SOCIETY REPORTS—

Richmond Academy of Medicine and Surgery........................................ 30

CORRESPONDENCE—

New York Letter. ........................................................................ 39

EDITORIAL—

The Anti-Vivisection Movement..... ............................. .................... 45

Patented Foreign, and Proprietary Domestic Preparations........................ 48

MEDICAL PROGRESS—

Comments upon Recent Obstetrical Literature........................................ 49

SELECTIONS AND ABSTRACTS........................................................ 55

MEDICAL ITEMS........... ............................................................ 62

BOOK REVIEWS.............................................................................. 65

MEDICAL ITEMS—Continued .......................................... x, xii, xiv, xv, xxvi

ADVERTISERS' INDEX TO ADVERTISEMENTS ........................................ xviii

CLASSIFIED INDEX TO ADVERTISEMENTS........ ................................xxxiii

